# Apes have culture but may not know that they do

**DOI:** 10.3389/fpsyg.2015.00091

**Published:** 2015-02-06

**Authors:** Thibaud Gruber, Klaus Zuberbühler, Fabrice Clément, Carel van Schaik

**Affiliations:** ^1^Department of Comparative Cognition, Institute of Biology, University of NeuchâtelNeuchâtel, Switzerland; ^2^School of Psychology and Neuroscience, University of St AndrewsSt Andrews, UK; ^3^Cognitive Science Centre, University of NeuchâtelNeuchâtel, Switzerland; ^4^Anthropological Institute and Museum, University of ZürichZürich, Switzerland

**Keywords:** animal culture, comparative cognition, field experiments, cultural mind, metarepresentation

## Abstract

There is good evidence that some ape behaviors can be transmitted socially and that this can lead to group-specific traditions. However, many consider animal traditions, including those in great apes, to be fundamentally different from human cultures, largely because of lack of evidence for cumulative processes and normative conformity, but perhaps also because current research on ape culture is usually restricted to behavioral comparisons. Here, we propose to analyze ape culture not only at the surface behavioral level but also at the underlying cognitive level. To this end, we integrate empirical findings in apes with theoretical frameworks developed in developmental psychology regarding the representation of tools and the development of metarepresentational abilities, to characterize the differences between ape and human cultures at the cognitive level. Current data are consistent with the notion of apes possessing mental representations of tools that can be accessed through re-representations: apes may reorganize their knowledge of tools in the form of categories or functional schemes. However, we find no evidence for metarepresentations of cultural knowledge: apes may not understand that they or others hold beliefs about their cultures. The resulting Jourdain Hypothesis, based on Molière’s character, argues that apes express their cultures without knowing that they are cultural beings because of cognitive limitations in their ability to represent knowledge, a determining feature of modern human cultures, allowing representing and modifying the current norms of the group. Differences in metarepresentational processes may thus explain fundamental differences between human and other animals’ cultures, notably limitations in cumulative behavior and normative conformity. Future empirical work should focus on how animals mentally represent their cultural knowledge to conclusively determine the ways by which humans are unique in their cultural behavior.

“Par ma foi! Il y a plus de quarante ans que je dis de la prose sans que j’en susse rien, et je vous suis le plus obligé du monde de m’avoir appris cela.” Mr Jourdain, Le Bourgeois-Gentilhomme, Acte II, scène 4, Molière (1670).

[“By my faith! For more than forty years I have been speaking prose without knowing anything about it, and I am much obliged to you for having taught me that.” Mr Jourdain, The Middle-class Gentleman, Act II, scene 4, Molière (1670) The Gutenberg Project, translation by Philip Dwight Jones].

## APE AND HUMAN CULTURES: A DIFFERENCE IN DEFINITIONS?

Over the last decades, numerous studies have provided evidence for culture-like phenomena in wild animals, especially great apes. Evidence is usually in terms of group-specific behavior patterns (Whiten et al., 1999; Rendell and Whitehead, 2001; Perry et al., 2003b; van Schaik et al., 2003), which sometimes involves neighboring groups that live in nearly identical environments and are genetically indistinguishable (Krützen et al., 2011; Gruber et al., 2012a; Luncz et al., 2012). Furthermore, there is good evidence that social learning is the primary force that explains differences between communities in a number of species as opposed to genetic or environmental factors (Jaeggi et al., 2010; Kendal et al., 2010; Allen et al., 2013; Samuni et al., 2014). Building on a long tradition of experimental work in non-primate species (Warner, 1988; Reader and Biro, 2010), a promising approach is to use experimental techniques in the wild (Matsuzawa, 1994; Biro et al., 2003; Gruber et al., 2009), such as seeding a novel behavior to investigate whether it spreads throughout a community (van de Waal et al., 2010).

Although much of this research is still on-going, many scholars now assume that some animal behaviors are ‘cultural’ because they are socially transmitted across generations, thus fulfilling the widely accepted definition of animal traditions as “a distinctive behavior pattern shared by two or more individuals in a social unit, which persists over time and that new practitioners acquire in part through socially aided learning” (Fragaszy and Perry, 2003, p. xiii). This definition of traditions would be sufficient to define culture for most biologists, but culture can also be fine-tuned as “the possession of multiple traditions, spanning different domains of behavior” (Whiten and van Schaik, 2007, p. 605). However, two sources of skepticism remain. First, an equally influential school of thought argues that ape ‘cultures’ result from convergent rather than homologous processes (Galef, 2009; Tomasello, 2009), mainly because of differences in the underlying social learning mechanisms found in humans and other great apes. As a result, apes may be incapable of producing cumulative cultural evolution. Second, for some authors culture is more than a conglomerate of socially acquired behaviors, and should therefore rather be defined as an integrated set of norms that its owners stand for and defend (Hill, 2009; Perry, 2009). Whether or not these defining aspects of human culture are also present in animals is currently unknown, which may explain why results from primatology so far have been seen as largely irrelevant by many in the social sciences (Hill, 2009; Perry, 2009).

In the following, we review what are currently considered the two major differences between ape and human culture – cumulative culture and normativity – and argue that differences in metarepresentational processes, the cognitive ability to generate representations of representations, underlie these ape-human differences, offering a general explanatory framework. While we acknowledge that it is possible to adopt lower-level explanations to analyze animal behavior, including that of great apes (Heyes, 1998; Shettleworth, 2010), we believe that there is compelling evidence that human culture originates from primate roots. Our goal in this article is to distinguish where exactly human and great ape cultures differ to precisely identify what evolved uniquely in the human lineage to generate modern human cultures.

## IMITATION, TEACHING AND CUMULATIVE CULTURE

The first line of argument for a discrepancy between human and animal culture concerns the nature of the underlying mechanisms of social learning. Only human culture, it is argued, results from imitation and teaching, while animal cultures are produced by ‘lower-level’ social learning, such as stimulus enhancement or emulation (Tomasello, 1990, 2009; Galef, 1992, 2009; Zuberbühler et al., 1996; but see Whiten et al., 2009). This is supported by the fact that there is generally no good evidence for teaching in non-human primates, in stark contrast to the habitual natural pedagogy found across human societies (Csibra and Gergely, 2009; but see Thornton and Raihani, 2010 for evidence of teaching in non-primate species). The role of imitation in animal culture, however, is more complex. Although chimpanzees can imitate (Whiten et al., 2007; Hobaiter and Byrne, 2010), it is not clear whether imitation plays any role in the transmission of behaviors (Whiten et al., 2009) or in maintaining traditions in nature (Claidière and Sperber, 2010).

Similar arguments have been made in relation to ‘cumulative culture,’ which emerges from individuals’ abilities to ‘ratchet’ existing culturally transmitted achievements, that is to “…add an existing technique used in a different context, or an entirely novel technique, to an existing technique, and integrate them functionally” (Pradhan et al., 2012, p. 181). Human culture is profoundly more cumulative than anything ever documented in animals, including apes. Composite tools, which are “made of at least two different material elements that are kept together so as to function as one tool” (Boesch, 2013, p. 31), are completely lacking in wild chimpanzees although they show evidence for basic cumulative phenomena (Matsuzawa, 1991; Sanz and Morgan, 2007; Boesch et al., 2009), as do captive chimpanzees (Yamamoto et al., 2013), captive orang-utans (Lehner et al., 2011) and New Caledonian crows (Hunt and Gray, 2003). In sum, more work is needed to confirm the cumulativeness of animal behavior.

Some argue that humans have cumulative culture because only we have both teaching and imitation (Galef, 2009; Hill, 2009; Dean et al., 2014) but human-animal differences in social learning mechanisms may not be sufficient to explain the emergence of cumulative culture (Yamamoto et al., 2013). Many components of human culture are causally opaque, that is, they cannot be understood or developed by a naïve individual without specific instructions or explanations by a knowledgeable individual. The development of a natural pedagogy to transmit this knowledge may thus have acted as the main force of cumulativeness (Csibra and Gergely, 2011; Pradhan et al., 2012). Animal cultures, in contrast, are argued to be causally transparent and to not require instructions to be acquired (Csibra and Gergely, 2011).

Cognitively, intentional teaching and especially pedagogy appear demanding, in that multiple representations must be stored, manipulated, and compared simultaneously (Gergely et al., 2007). We therefore argue that higher levels of cumulative culture depend on representational abilities, to be examined in detail below.

## FROM INFORMATIVE TO NORMATIVE CONFORMITY

A more recent line of argument for a qualitative difference between ape and human culture is based on the notion of ‘conformity’ (Whiten et al., 2005; Claidière and Whiten, 2012; van de Waal et al., 2013; van Leeuwen and Haun, 2013). The term was originally defined as the alignment of one’s attitude with a majority position (Asch, 1956; Cialdini and Goldstein, 2004), a ‘majority influence’ (van Leeuwen and Haun, 2013). Recent studies suggest that conformity-like phenomena may also exist in animals, even to the point of forsaking a pre-existing individual preference for the majority’s preference (Whiten et al., 2005; Hopper et al., 2011; Claidière and Whiten, 2012; van de Waal et al., 2013). However, the underlying cognitive mechanisms of these behavioral effects are largely unknown, particularly whether animals are simply biased to select the choice of the majority (*informational conformity*) or whether this is the result of social awareness and a desire to conform to the group (*normative conformity*; Deutsch and Gerard, 1955; Claidière and Whiten, 2012; van Schaik, 2012).

Importantly, while both mechanisms occur in humans, there is currently no good evidence for normative conformity in animals. In humans, normative conformity is demonstrated if individuals are less likely to choose the behavioral variant of the majority in private than social contexts (Deutsch and Gerard, 1955), a paradigm that to our knowledge has not yet been used with non-human primates. An open question remains how important majority influences really are in the transmission of animal behavior, as most empirical studies have not quantified differences in social transmission rates as a function of the number of available models (van Leeuwen and Haun, 2014), and whether there really exists a disproportionate tendency to copy the majority in non-humans. Another good indicator for normative conformity is the punishment of individuals who deviate from social norms (Hill, 2009, p. 276). In the animal behavior literature, the term ‘punishment’ usually refers to a retaliatory action that leads to future compliance by the punished individuals (Clutton-Brock and Parker, 1995). Certain processes are shared by both informational and normative conformity (van Schaik, 2012), with informational conformity forming the basis for normative conformity. A graded integration of already present underlying mechanisms, such as informational normativity, fairness-related behaviors (Brosnan, 2013) or punishment, may have thus led to normative conformity.

Similar to what has been argued for cumulative culture, graded cognitive differences may explain the jump from informational to normative conformity. Normativity requires some representation of norms and its more complex expressions therefore will also depend on the extent to which representations can be stored, manipulated and compared (Kaufmann and Clément, 2014). This leads to the suggestion that, from a proximate mechanistic perspective, the ability to access the representational content of one’s knowledge may represent the critical difference between humans and other species. The analysis of the representational dimension of culture requires a cognitive approach, which we will develop in the next sections.

## A COGNITIVE APPROACH TO THE STUDY OF CULTURE

Psychological studies of humans have repeatedly documented how culture affects cognition (Mesquita and Frijda, 1992; Greenfield, 1997; Kitayama et al., 2003; Sperber and Hirschfeld, 2004; Nisbett and Miyamoto, 2005) in domains as diverse as spatial cognition (e.g., Levinson, 1992; Levinson et al., 2002), behavioral economics (Henrich et al., 2005), or time perception (Casasanto, 2008). For instance, children initially prefer geocentric (absolute) strategies in spatial memory tasks but by age eight show culturally dependent strategies, which is also reflected in their spatial language (Haun et al., 2006). Additionally, the same study showed that great apes also prefer geocentric strategies, suggesting a shared evolutionary origin. However, despite such studies and despite considerable interest in the cognitive underpinnings of animal social behavior (Call and Santos, 2012), less work has been conducted to understand how cognition and culture intertwine when it comes to representing knowledge in non-humans. As a result, the human-animal gap remains wide, with animal cultures characterized by group-specific catalogs of behaviors and human cultures characterized by group-specific catalogs of norms and their practices.

Nonetheless, one often quoted definition of culture in the animal culture debate is “the way we do things” (McGrew, 2004). This requires an ability not only to mentally represent behaviors, but also to identify the majority default behavior and compare this with one’s own behavior. Humans are certainly endowed with the ability to analyze their and others’ behavior, which enables them to represent what they and others know and to define themselves in terms of cultural groups. We can define this ability as ‘thinking culturally’; but is there any indication for this in apes? Most animal studies have not attempted to address the extent to which mental representations affect cultural behavior.

**FIGURE 1:**
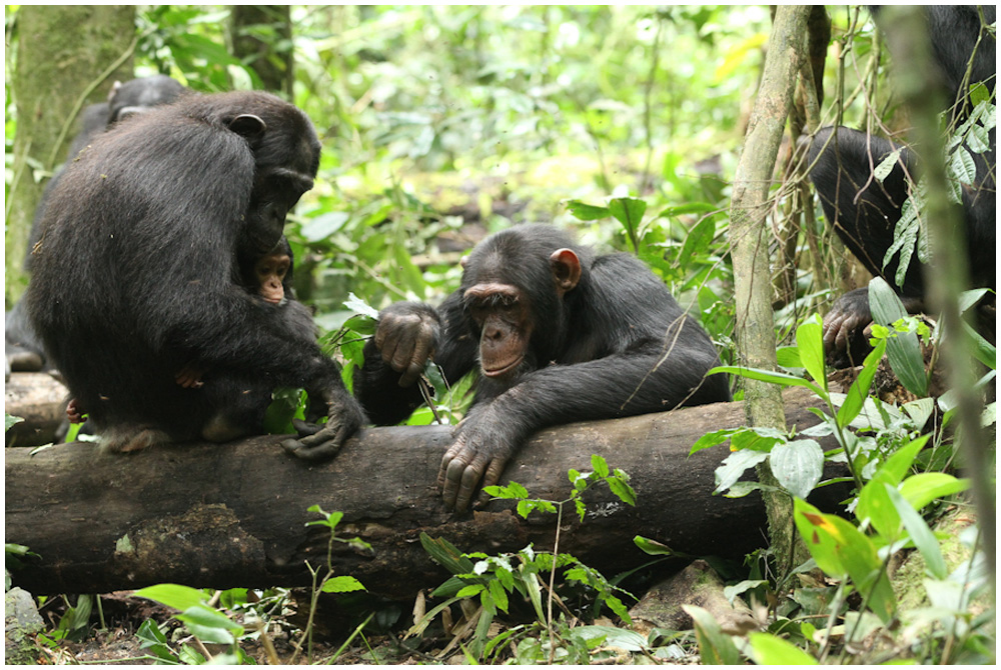
**A member of the Kanyawara community extracting honey from the honey-trap apparatus during an experimental trial.** (Kibale National Park, Uganda, Courtesy of Andrew Bernard).

One way to address the cognitive processes underlying animal culture empirically is to present individuals belonging to different cultural groups with a problem that can be solved in different ways. If the problem is solved in line with pre-existing behavioral preferences, then this can be interpreted as a signal for differences in underlying mental representations. This interpretation is particularly compelling when individuals do not seem to comprehend their environment in the same way, notably if one object (such as a stick) appears to be understood *as* a tool in one given community, but not in the other one. Possessing mental representations defines the ability to think (Byrne, 1995); being able to access and modify these representations is a crucial feature to cope with everyday tasks. However, species may differ in their capacity to do so. In a recent example, two groups of chimpanzees in Uganda, the Sonso community of Budongo Forest and the Kanyawara community of Kibale Forest, were exposed to an identical problem, honey trapped in a cavity of a large tree trunk (**Figure 1**, Gruber et al., 2009). The two communities differ culturally, especially in terms of whether or not they use sticks as foraging tools (Whiten et al., 2001). Results showed that members of the two communities solved the problem with group-specific techniques consistent with their cultural knowledge, that is, stick use in Kanyawara and leaf-sponging in Sonso. Hence, the chimpanzees applied previously acquired tool use behavior to a novel foraging problem. A particularly relevant point was that, although all Kanyawara chimpanzees knew how to manufacture leaf-sponges, no one chose this technique.

In a follow-up experiment, individuals were exposed to the same problem, honey trapped in a cavity, but were also provided with a multi-functional tool, the ‘leafy-stick,’ which could be transformed into at least three different tools: a sponge, stick, or brush. Results showed that members of the two communities found different parts of the tool salient and used them accordingly. At Sonso, individuals detached the leaves from the provisioned tool to manufacture a leaf-sponge, while at Kanyawara, the chimpanzees used the stick part of the tool to dip for honey (Gruber et al., 2011).

Overall, these experiments suggest that chimpanzee behavior is determined by previous experience, or knowledge, which can differ between communities (Gruber et al., 2009). As a result, different communities may differ in how they recognize and use the affordances of an identical tool, suggesting that the way chimpanzees perceive their environment is biased by cultural knowledge (Gruber et al., 2011).

In a related study, rehabilitant orang-utans (wild-born ex-captive individuals living in a sanctuary before reintroduction into the wild) were exposed to the same honey-dipping task and to a raking task. When individuals from two genetically distinct populations were tested they showed no difference in their performance in the raking task, suggesting that their potential understanding of sticks as tools was similar. In contrast, in the honey-dipping task, their performance varied in line with whether or not stick use was prevalent in their native populations, thus replicating the findings in chimpanzees (Gruber et al., 2012b). Recently, the same patterns have been reported from two populations of capuchin monkeys (*Sapajus apella*): monkeys that were naturally unfamiliar with manufacturing stick tools ignored and even discarded stick tools that were provisioned to gain access to an experimentally provided food source, while capuchins familiar with stick tool manufacture used sticks to obtain the same food (Ottoni, personal communication). Overall, these results are consistent with the view that, across primates and including humans, cultural knowledge influences the way individuals perceive their environment and how they act on it, in line with the notion of ‘cultural affordances,’ the “opportunities for perception and action offered by the environment to an organism” (Kaufmann and Clément, 2007, p. 226).

**FIGURE 2:**
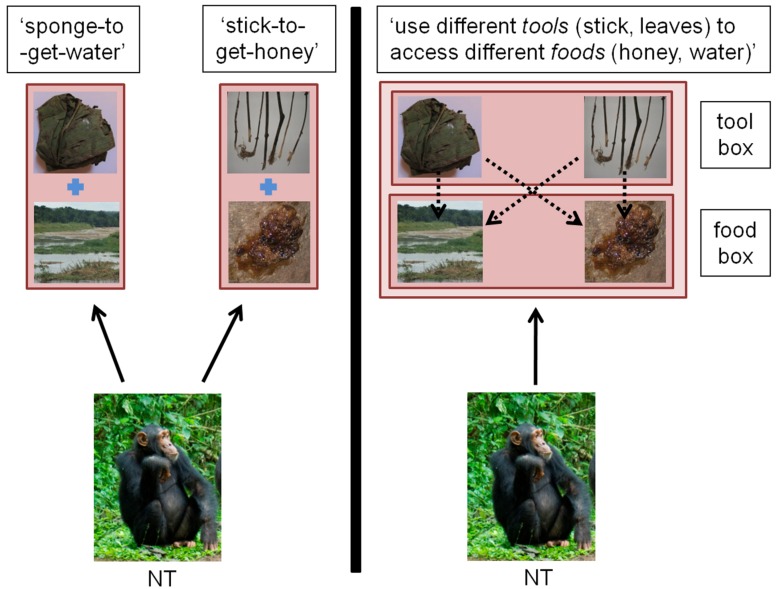
**Comparison of a representational system where individuals build independent representations** (A)** or can re-organize their knowledge into categories **(B)** in the case of tool use.** Full arrows: act of mentally representing. Square: content of mental representation, with or without embedded representations. Dashed arrows: connections within or between mental representations. **(A)** *Independent Representations*: individual NT forms a learned association between distinct parts of the environment (for example, a stick is associated with obtaining honey; a leaf-sponge is associated with obtaining water). The resulting two mental representations are independently stored in the individual’s mind. **(B)** *Re-organization of knowledge in categories*: individual NT organizes individual representations hierarchically, potentially under larger object kinds. For instance, ‘sponges’ and ‘sticks’ become members of the more general concept ‘tools’ in the individual’s own representational system and can be used interchangeably onto the different substrates ‘honey’ and ‘water.’ In the wild, chimpanzees are known to use leaf-sponges to fetch water, a behavior present in all studied communities. Additionally, in some communities, chimpanzees have been observed to use sticks to access liquid, a behavior named ‘fluid-dip’ (Whiten et al., 2001). (Photos of honey, stick, and river by Thibaud Gruber; photos of chimpanzees and leaf-sponge, courtesy of Nina Hänninen and Cat Hobaiter).

In spite of these results, an important unresolved question still is whether the mental representations underlying such variations are akin to ‘cultural ideas’ (or cultural mental representations), that is, mentally “stored form[s] derived from experience […] used to generate actions,” (Bryson, 2009, p. 83). Here, we define a mental representation as an *internal cognitive construction of the mind that represents an aspect of the world*. In doing so we follow Leslie (1987, p. 414) who assumes that “…the basic evolutionary and ecological point of internal representation must be to represent aspects of the world in an accurate, faithful and literal way, in so far as this is possible for a given organism. Such a basic capacity for representation can be called a capacity for primary representations. Primary representation is thus defined in terms of its direct semantic relation with the world.” For instance, in the context of tool use, the idea of ‘stick use’ could be defined as a mental representation that contains the object ‘stick’ and some of its functional properties, which are defined in terms of specific actions. In other words, a learned association between a tool and a reward can be represented as a unique mental representation (e.g., ‘stick-to-get-honey,’ **Figure 2A**). This mental representation can be cultural because it can be wildly shared within the members of a given community, as the behavior it represents (Sperber, 1996).

Great apes are cognitive animals (Call and Tomasello, 2008), in the sense that they can store their knowledge as primary representations, but the key question is whether they also have more complex representations, for instance, to represent and classify an object (e.g., stick) as belonging to a broader class (e.g., tool; **Figure 2B**), and not solely attached to a given reward as in the ‘stick-to-get-honey’ mental construction. In the following, we apply conceptual tools of developmental psychology to analyze the complexity of mental representations underlying great ape cultural behavior.

## MENTAL REPRESENTATIONS OF ARTIFACTS: A DEVELOPMENTAL PERSPECTIVE

Developmental psychology has long been interested in how infants come to understand their environment and the objects found therein (Piaget, 1929), including artifacts (Margolis and Laurence, 2007). ‘Artifacts’ are a special class of objects because they have been modified or created for a specific purpose, and are thus major components of human material culture. Two-year-olds appear to understand object kind and some artifactual properties (for instance, they give an appropriate response to ‘a’ tool or ‘a’ musical instrument) but do not yet have an overall concept of tools (Mandler, 2007). Three-year-olds, however, start to understand that artifacts belong to higher-order units, characterized by the purpose they are ‘made for’ (DiYanni and Kelemen, 2008). This important cognitive and representational shift, which occurs between age five and seven, is characterized by a transition from a vague to a well-defined understanding of an artifact’s function and typical or intended use (Defeyter and German, 2003).

Empirically, this shift can be demonstrated by what has been termed ‘*functional fixedness’*: children experience difficulties in solving a problem because of interference by previous knowledge. For instance, children may fail to see a solution to a tool-use problem if they are being offered a tool presented in a situation where it already has a well-defined purpose but where the situation requires a different use of this tool (Defeyter and German, 2003). That children before age five do not show functional fixedness may be because they do not represent the intentionality of the maker, failing to understand that a tool has been intentionally manufactured by a designer to fulfill a specific function, a phenomenon known as adopting a *design stance* (German and Defeyter, 2000; Kelemen and Carey, 2007). However, other interpretations of functional fixedness exist and do not connect it to the design stance. Individuals may simply fail to see multiple uses of an object because previous experience has led them to form an association between an object and a given function. Hence, the function itself is not represented as ‘intended.’ This interpretation has been argued for captive chimpanzees (Hanus et al., 2011) and may explain why in the honey-trap experiments discussed before, chimpanzees failed to use sticks, mainly because this material is used daily to build nests, which may have prevented them from considering sticks as tools to extract honey. This interpretation obviously makes functional fixedness a less cognitively complex mechanism, but other wild chimpanzee communities have overcome any fixedness on nest-building by having learned to incorporate sticks into their extractive tool repertoire. This observation argues against the ‘simple’ functional fixedness hypothesis. This idea faces another problem when applied to the honey-trap experiment. It is unable to explain how the Sonso chimpanzees disregarded their only known function of leaf-sponges (to absorb drinking water) in favor of extracting honey from experimental cavities (Gruber et al., 2011). This suggests that leaf-sponges are not functionally fixed to the purpose of extracting water, although experiments presenting water and honey simultaneously are needed to support this hypothesis. It is unlikely that the chimpanzees simply mistook the honey for water because it was very obvious during the experiments that subjects were aware that the resource was honey and not water, often visibly reacting to the stickiness of honey by rubbing their hands on the logs. Moreover, no individuals at Kanyawara made a sponge to extract the honey, despite leaf-sponging being customary in this community, suggesting that the confusion hypothesis can be rejected. Therefore, it is more plausible that the Sonso chimpanzees produced leaf-sponges to extract honey by some form of analogical reasoning (Gillian et al., 1981), a cognitive process that requires access to representational content (Gentner and Markman, 1997): they may either have considered that all liquids should be treated the same, or, reversely, that a leaf-sponge may be used on different liquids.

In summary, functional fixedness remains a possible explanation for the patterns observed in wild and captive chimpanzees, although it is difficult to decide whether this is based on simple or complex processes. Thus, it remains possible that chimpanzees access their mental representations in a more active way, akin to early reports of ‘insight’ (Köhler, 1925). At the very least, individuals must have activated a mental representation (e.g., leaf-sponge) without the corresponding real world experience that generated the representation in the first place (waterhole). For example, a chimpanzee may think of a leaf-sponge when finding a valuable resource in a cavity, without seeing an actual leaf-sponge – it can start looking for the appropriate leaf material to manufacture one as a consequence. Accessing knowledge, however, may be cognitively more complex and may require that the subject also knows that it has the knowledge of leaf-sponges, which requires the ability to generate representations of representations, i.e., metarepresentations (Sperber, 2000b). In conclusion, while ‘simple’ functional fixedness appears to act without actively accessing representations (that is, individuals do not need to be aware of the content of their knowledge), ‘complex’ functional fixedness, as seen in older children, is based on representing intentions (a design stance), a form of metarepresentation.

To facilitate progress regarding the relation between chimpanzee and human culture, we will next survey the different levels of representation that may or may not underlie ape cultural behavior. Our focus will be on processes that can be described as ‘metarepresentational’ in the context of culture.

## METAREPRESENTATIONS AND CULTURE

The ability to attribute psychological states to others and to oneself, or to have a ‘theory of mind,’ has been identified as the most important cognitive difference between humans and animals, including great apes (Premack and Woodruff, 1978; Call and Tomasello, 2008). The classic test for investigating an individual’s theory of mind is the ‘Sally–Anne’ test, a false-belief task. In its standard version, the subject is presented with a performance during which an agent (a doll) witnesses how an object is being placed in box A, but then is prevented from seeing how the object is moved to another box B. As a result, the agent will have a false belief about where the object is hidden, i.e., in box A rather than box B. Human infants generally understand such tasks from age four (Doherty, 2008), although more recent research has shown that precursor abilities required to solve the task emerge as early as age two (Baillargeon et al., 2010).

Having a theory of mind requires some form of metarepresentational ability, the capacity to generate a representation of a representation. There is an ongoing debate about what exactly should qualify as a metarepresentation and different authors have proposed different terminologies. One important distinction has been to conceptualize metarepresentations either as ‘representations of representations’ (sense 1; e.g., Leslie, 1987) or as ‘representations of representations *as* representations’ (sense 2; e.g., Perner, 1991). In the latter case, the agent engaging in metarepresentation must represent the fact that whatever is represented is itself a representation. The paradigmatic example is the false-belief task, where the subject must understand that the agent’s representation of the world is ‘only’ a belief (object in box A), which differs from reality (object in box B). However, others have suggested that the subject does not necessarily have to be aware of the representational nature of the representation (Leslie, 1987). Perner (1991) uses the term ‘*secondary representation*’ to refer to sense 1 metarepresentations, as opposed to sense 2 metarepresentations, or ‘true’ metarepresentations.

Whiten (2000) has proposed a useful terminological distinction between the two senses, keeping the name ‘metarepresentation’ for sense 2 and describing sense 1 as ‘re-representations.’ In doing so, he uses a term introduced by Karmiloff-Smith (1992) as a cognitive mechanism that allows accessing and sorting acquired information: “...a way to gain knowledge is for the mind to exploit internally the information that it has already stored (both innate and acquired), by redescribing its representations or, more precisely, by iteratively re-representing in different representational formats what its internal representations represent” (ibid., pp. 15–16). This individual-centered definition was subsequently extended to social processes by Whiten (2000), who found evidence that enculturated apes engage in ‘re-representation’ during imitation and pretense. In sum, the term ‘re-representation’ (sense 1 metarepresentation, secondary representation) commonly describes a metarepresentation that does not require its owner to be aware of the representational nature of its representation, while the term ‘metarepresentation’ (sense 2 or true metarepresentation) requires an awareness of the representational nature (**Table 1**), a terminology that we also adopt in this paper. Additionally, the wording ‘metarepresentational processes’ will describe the general ability to go beyond simple representations, that is to engage at least in sense 1, and possibly in sense 2 metarepresentations.

**Table 1:** Connection between Metarepresentation Sense 1 and Sense 2, the context, individually centered or socially oriented, in which they occur; and the way they have been described in the literature.

Sense		Metarepresentation sense 1	Metarepresentation sense 2
Context		Representation of representation	Representation of representation *as* a representation
Individually centered		Re-representation (*sensu* Karmiloff-Smith, 1992)	Representation of one’s own beliefs as beliefs (*sensu* Carruthers, 2009)
Socially oriented		Ape-like theory of mind (Re-representation *sensu* Whiten, 2000)	Full-blown theory of mind (*sensu* Perner, 1991)

In the following section, we review the different metarepresentational processes which appear central to the representation of tools, and more generally to culture, and order them in a way that could constitute an evolutionary pathway. Our goal is to identify the different types of representations and metarepresentations that could underlie and sustain animal cultures^1^.

### RE-REPRESENTATIONS TO FACILITATE CATEGORISATION AND CONCEPTUALISATION

Group differences in tool use behavior importantly contributed to the claim that chimpanzees have culture (Whiten et al., 1999). Nevertheless, it is still unclear whether chimpanzees and other tool-using primates resemble human infants (Träuble and Pauen, 2007) in having a true understanding of ‘kinds,’ such as tools, or types of tools, such as hammers (Hauser and Santos, 2007; Hernik and Csibra, 2009). Therefore, understanding how primates mentally represent tools is key for any comparison between human and ape cultures. Humans are often considered unique in their ability to represent objects as ‘made for something,’ that is, to naturally adopt a teleological stance when dealing with them (Gergely and Csibra, 2003; Ruiz and Santos, 2013), an ability that appears to facilitate the acquisition of tool use behavior by toddlers (Hernik and Csibra, 2009).

Not much theoretical work of this kind has been done in animals, despite the fact that great apes and capuchins are promising species to investigate these questions. Jackendoff (1989) argued that possessing a ‘true’ concept of something requires the ability to verbalize it. By this criterion, primates clearly lack the concept of ‘tool,’ but Jackendoff’s (1989) criterion may be unnecessary. In all likelihood, modern human language is a fairly recent evolutionary invention that emerged well after humans had developed complex and variable tools (Mithen, 1996). Thus, a conceptual system of images, which may also be available to non-linguistic species, may well have preceded a conceptual system of words (Gärdenfors, 2006).

An important question is whether animals can represent tools at a conceptual level (that is representing tools as objects with a given function to act on other objects) and not solely at a perceptual level (that is representing a tool based on its physical properties, Mandler, 2000). For example, can a chimpanzee categorize a leaf-sponge not only in terms of its perceived features (wadge of folded leaves) but also in terms of its function or purpose (liquid-absorption)? One possible way to investigate this question is to study whether apes classify novel objects according to functional (i.e., intended use) or perceptual similarities with familiar objects, similar to earlier paradigms developed to study analogical reasoning in chimpanzees (Gillian et al., 1981). Work with cotton-top tamarins and rhesus monkeys has shown that individuals can group objects into meaningful categories, such as tools, foods, animals or landmarks, as well as recognize distinctive features of tools (see Hauser and Santos, 2007 for a review). And for New Caledonian crows, it has already been shown that individuals can sort objects according to function, e.g., as sinking versus floating devices (Taylor et al., 2011; Jelbert et al., 2014). Considering these results, it appears likely that tool-using primates such as chimpanzees, orang-utans or capuchins represent their tools as particular objects with a function to act on other parts of their environment, that is, at the conceptual level, but experimental work is needed to confirm this hypothesis.

The main benefit of re-representations is that they allow their bearer to reorganize acquired information, for example by allocating objects to categories, such as a leaf-wadge to a sponge tool. **Figure 2** illustrates this process in the context of tool use as a shift from a simple to a complex representational format. In the simple representational format, each tool is mentally represented as having one purpose (e.g., ‘sponge-to-get-water’) with no connection between representations. In the complex representational format, simple representations also belong to more general categories, and the items belonging to one category can be selected to function on the items belonging to a different category [e.g., ‘use different tools (stick, leaves) to access different foods (honey, water)’]. One relevant observation here is that in the Gruber et al. (2011) study, the Sonso chimpanzees spontaneously used leaf-sponges to extract honey, although this tool is widely used by wild chimpanzees for no other purpose than to extract water from streams or cavities (Whiten et al., 1999). One interpretation of this finding is that leaf-sponges are not exclusively and rigidly represented in connection with water, suggesting that the Sonso chimpanzees have employed re-representational abilities to find this solution. Nonetheless, because they appear to fail to consider sticks as potential tools in other experiments, their re-representational abilities may only allow some flexibility around already known artifacts but may be too limited to generate the general concept of ‘tool.’

### RE-REPRESENTATIONS AS REPRESENTATIONS OF TECHNIQUES

**FIGURE 3:**
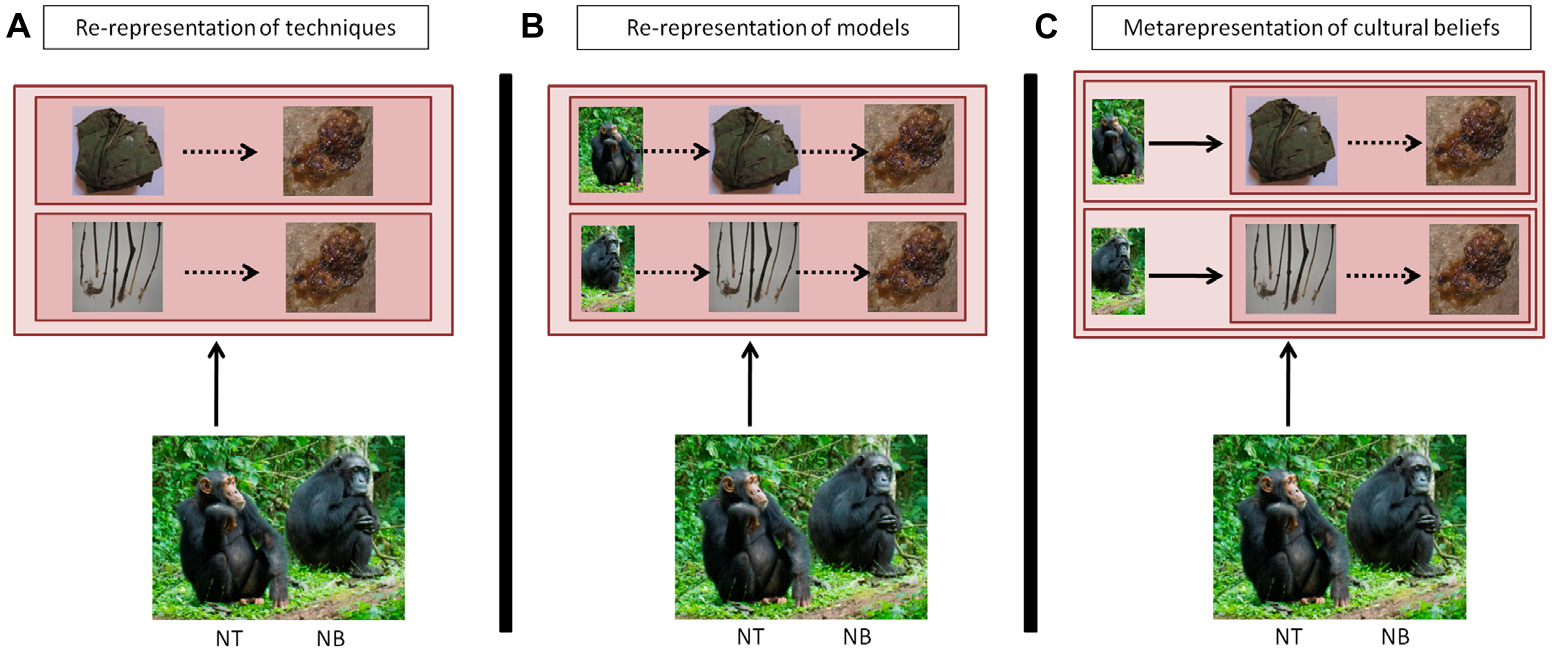
**Comparison of a representational system where individuals can re-represent in parallel several actions in their mind **(A)**; can re-represent the identity of the individual attached to the representation of the tool-using activity **(B)**; and can fully metarepresent that other individuals may have different beliefs than themselves, applied to tool use **(C)**.** Full arrows: act of mentally representing. Square: content of mental representation, with or without embedded representations. Dashed arrows: connections within or between mental representations. **(A)** *Re-representations of techniques*: individual NT represents in parallel two different ways to achieve the same outcome: ‘obtaining honey.’ Re-representations allow individual NT to access and compare both techniques, to select the most suitable one to achieve the task. **(B)** *Re-representation of techniques displayed by individuals*: individual NT represents in parallel that she is obtaining honey by using a leaf-sponge, while individual NB is obtaining the same resource with a different tool, a stick. **(C)** *Metarepresentation of others’ cultural beliefs*: here, individual NT knows that she has certain beliefs, for instance that honey should be gathered with a leaf-sponge; she also knows that other individuals such as NB possess systems of beliefs – that is, individual NT represents that individual NB represents that… – and also represents the fact that the content of these beliefs may be similar or different from her own. In the illustrated case, individual NB knows that the best way to obtain honey is by using a stick, which differs from NT’s information, which may result from individual or cultural learning. (Photos of honey, stick and river by Thibaud Gruber; photos of chimpanzees and leaf-sponge, courtesy of Nina Hänninen and Cat Hobaiter).

A second characteristic of re-representations is to allow an individual to maintain multiple mental representations simultaneously. During imitation, for instance, an individual may hold representations of an action’s desired outcome and an effectively executed motor pattern to achieve it (Whiten, 2000). Similarly, an individual may be able to simultaneously maintain separate mental models of two actions in order to compare them (Perner, 1991). In the case of ape tool use, for example, re-representations may allow an individual to generate representations of competing techniques and compare them to solve a problem (e.g., representations of leaf-sponging and stick-using to obtain honey; **Figure 3A**). A recent study from the Sonso chimpanzee community is in line with this interpretation. In November 2011, a few individuals discovered a novel tool behavior, moss-sponging, to access water from a natural clay hole (Hobaiter et al., 2014). Importantly, all moss-using individuals were already skilled leaf-sponge makers, suggesting that they possessed mental representations of two techniques for one outcome, accessing clay water, or that they modified their existing mental representation of a leaf-sponge to add the possibility of moss instead of leaves, in contrast to others who did not develop the novel behavior. Whether or not individuals also compared both representations cannot be decided by this study. Interestingly, the two techniques differ in efficiency (moss-sponges appear to hold water better than leaf-sponges), suggesting that individuals should prefer moss-sponging, whenever moss is locally available. Primates are capable of assessing the physical properties of their tools, in particular with respect to size and weight of potential objects that can be used as tools to complete a task (Matsuzawa, 1994; Fox et al., 1999; Visalberghi et al., 2009). However, there is also evidence for cultural conservatism and individual habit formation in primates, which may prevent them from changing techniques (Hrubesch et al., 2009; Gruber et al., 2011; Brosnan and Hopper, 2014). More field experiments are needed to address how chimpanzees and other animals evaluate the efficiency of their techniques. If chimpanzees choose a solution that is more efficient than a habitual technique already present in their repertoire, a stronger association between the novel tool and the original substrate may be formed, leading ultimately to a change in the tool choice. However, this may require several trials to be achieved, which may not always be granted in natural settings. The ability to compare mental representations, allowed by re-representational abilities, may allow switching to the novel technique directly after one individual trial or after witnessing others display this technique during social learning, making it a much faster, potentially more adaptive, process.

### RE-REPRESENTATIONS AS REPRESENTATION OF INDIVIDUAL DIFFERENCES

A more complex form of re-representation is for an individual to understand that it is carrying out one technique while others may carry out the same or a different technique, albeit toward the same goal; or the individual may also represent itself performing two different techniques. Here, the re-representation is not only functional but also has a social dimension, by means of comparing the self with others or between others. This mechanism may underlie the perception of intergroup differences, if such observations are possible. In chimpanzees, this will be rarely the case because of the species’ intense intergroup hostility (Herbinger et al., 2009; Wilson et al., 2014) but this may be studied in dispersing adolescent female chimpanzees who adopt their new community’s cultural practices (Luncz and Boesch, 2014). It would also be interesting to investigate how bonobos, who tend to be much more tolerant to the presence of strangers (Furuichi, 2011), would react to the sight of other communities displaying different behaviors from themselves. One prediction thus is that normative aspects of culture will only appear in species where close intergroup interactions are common, a necessary precondition for the evolution of group-mindedness as a psychological trait (Gruber and Zuberbühler, 2012; Tomasello et al., 2012). Equally relevant are imitation tasks, in which chimpanzees of high social status tend to be preferred as models for new techniques; however, this may also result from the fact that they are generally more attended to (Horner et al., 2010; Kendal et al., 2014). In Hobaiter et al.’s (2014) study, social learning was very effective for moss-sponging, first shown by the alpha male, but not very effective for leaf-sponge re-use, first shown by lower-ranking individuals. Overall, these findings suggest that information on the identity of the model is part of an individual’s representation of a technique (**Figure 3B**).

### RE-REPRESENTATIONS AS A BASIS FOR THE CUMULATIVENESS ASPECT OF CULTURE

A final characteristic of re-representations is that they allow representing tools, techniques, and the function of each component or sequence of actions (Byrne and Russon, 1998) and their outcomes. They therefore allow representing a tool independently from the functional scheme in which it was originally defined. The ability to hold several representations in one’s mind at the same time and compare them may help an individual to associate different parallel functional schemes with complementary outcomes, thus leading to the creation of a novel scheme combining the two former ones. For instance, to build a composite tool, it is necessary to represent that both components can afford the same action, but also that their association results in higher efficiency. The Acheulean hand-ax, observed from about 1.7 million years ago, may have required well developed re-representational processes to form the necessary mental template (Mithen, 2007; McPherron, 2013).

The near-absence of cumulative culture in apes, may therefore be due to their limited re-representational abilities, allowing some flexibility around behaviors that are already present in the repertoire (for instance to invent a moss-sponge based on a known leaf-sponge) but making qualitative jumps very unlikely. In contrast, belief-based metarepresentations do not seem crucial to analyze the functional schemes present in one’s current knowledge and to seek how to improve them. Re-representations may also sustain other complex cognitive processes recently proposed to be involved in the cumulativeness of human culture such as mental time travel (Fogarty et al., 2012; Vale et al., 2012). There is evidence for mental time travel coming from a range of other animals than humans, including great apes and corvids (e.g., van Schaik et al., 2013), although alternative explanations have been proposed (Fogarty et al., 2012; Vale et al., 2012). This suggests that some re-representational abilities are present in these species but that their extent is limited. In sum, more work is needed to precisely understand the scope of re-representations and their use in animals.

### METAREPRESENTATIONS TO REPRESENT OTHERS’ CULTURAL KNOWLEDGE

The highest stage of metarepresentational process, in our context, is to appreciate that members of another group may harbor beliefs that are different from one’s own group, that is, to compare ‘how things ought to be’ (**Figure 3C**). Here, cognition goes beyond simple re-representations, which could sustain all previous aspects of cultural knowledge, i.e., categorisation, representation of techniques, and representation of models. In effect, the metarepresentational processes must become ‘representations of representations *as* representations’ (*sensu* Perner, 1991, see **Table 1**), that is metarepresentations. In humans, this type of metarepresentation probably underlies complex mental state attribution, intentional teaching and belief-based imitation, the human ‘theory of mind’ (Tomasello et al., 2005 and comments; Meltzoff, 2007). The ability to mentally represent and compare own and others’ knowledge may refine the categorisation of partners as ‘same’ or ‘other.’ Such reasoning, if associated with feelings of group identity, appears to be an ingredient for the emergence of social norms. Humans have an urge to conform to the behavior of others, but to perceive group behavior as normative and recognize deviation, it is also necessary to mentally represent the group norm, ‘the way things ought to be.’ Humans tend to become aggressive toward non-followers, while positive reinforcement also plays a role, for instance, in the case of the ‘chameleon effect,’ when individuals engaged in an interaction unintentionally match each other’s behaviors (Chartrand and Bargh, 1999). How this effect connects to norms, however, remains to our knowledge to be investigated.

The theory of mind of great apes, in contrast, appears to be more limited and unable to take into account others’ false beliefs, suggesting that their metarepresentational abilities are equally limited (Call and Tomasello, 2008). Chimpanzees have access to others’ perceptual knowledge (Hare et al., 2000, 2001), but appear to have great difficulties accessing others’ beliefs, especially if they deviate from their own (Kaminski et al., 2008; Krachun et al., 2010). However, a research program studying how apes assess their own and others’ cultural knowledge has yet to be implemented. This research may also benefit other areas of metarepresentation. Controlled learning experiments (e.g., Marshall-Pescini and Whiten, 2008) will help to determine whether knowledgeable chimpanzees adjust their behavior to the state of knowledge of their naïve observers. During teaching, representing both the current state of knowledge of naïve individuals at the time of learning and the desired state of knowledge they must reach before being considered knowledgeable (e.g., they have acquired a set of rules or a technique) is crucial for knowledge transmission. In this respect, the ability to evaluate the state of knowledge of naïve observers, relying on metarepresentations, has most probably contributed to develop the full-fledge pedagogy now displayed by modern humans (Csibra and Gergely, 2009, 2011). While this remains to be tested, it is therefore unlikely that a chimpanzee would be shocked when it sees another behaving differently from the group norm, which would require the individual to understand that others may have values that deviate from its own and, by extension, to have a profound understanding of its own culture. Apes, in other words, are unlikely to consider whether ‘this is the right way to do it,’ a crucial feature of the human cultural mind and normative culture.

In sum, metarepresentations allow individuals to represent both one’s own cultural beliefs and those of others (Sperber, 2000a). Currently, we do not know whether this ability is present in apes and thus whether ape and human cultures differ in this important domain (see **Table 2**). As of now, the empirical data are consistent with what we call the ‘Jourdain Hypothesis,’ which states that apes have culture, but do not know that they do, in analogy to Molière’s Monsieur Jourdain, who had been speaking prose for 40 years without realizing it. By analogy, chimpanzees may practice their culture without knowing that they do, and future research must assess whether they can represent their own and others’ cultural behaviors as cultural norms. In the previous sections, we have shown that the ability to represent one’s own knowledge and others’ knowledge and the different ways of doing so appear fundamental to the human form of culture (e.g., Henrich et al., 2010). It thus appears necessary to determine whether a ‘Jourdain-like culture,’ that is, a culture its holder does not represent, can qualify as ‘culture,’ in particular with respect to other hominoids and to our earliest hominin ancestors, whose mental abilities may have been similar to those of great apes (Mithen, 1996).

**Table 2:** Summary of the different stages of representations involved in the cultural process and their presence in humans and great apes, according to current knowledge.

Representational stage		Humans (e.g., Sperber, 1996)	Non-human great apes
Species		
Primary (simple) mental representation		Present	Present (e.g., spatial memory, see Janmaat et al., 2013).
**Re-representations**
– Categorisation		Present	Present at the perceptual level but experiments needed to explore the conceptual level (Hauser and Santos, 2007; Ruiz and Santos, 2013).
– Representation of techniques		Present	Potentially present (Whiten, 2000; Hobaiter et al., 2014) but experiments needed to confirm their extent.
– Representation of practitioners		Present	Understanding of different models (Hopper et al., 2011) group identity present but no group-mindedness (Gruber and Zuberbühler, 2012; Tomasello et al., 2012).
Metarepresentation of cultural beliefs		Present	Absent (Call and Tomasello, 2008).

## SUMMARY AND FUTURE DIRECTIONS

The notion of metarepresentation has become widely used in false-belief research, while other metarepresentational processes have been neglected (Sperber, 2000b). Similarly, in the animal culture discussion, the latter have only played a role under the rubric of mindreading in comparing social learning processes that can lead to behavioral traditions, namely imitation and teaching (Tomasello et al., 2005 and comments). In this article, we have argued that metarepresentational processes may be useful to explain a wider range of features of human and animal cultures, from representing objects as culture-specific, meaningful artifacts to understanding that another individual may or may not share a cultural belief. For animals, it is conceivable that social learning acting on innovations driven by the environment is sufficient to generate the full range of behavioral traditions currently documented, but several recent studies question this hypothesis (van de Waal et al., 2013; Luncz and Boesch, 2014). In humans, however, culture is a co-construction of minds, and this may require considerable flexibility in how knowledge is organized. Therefore, culture without metarepresentational processes may never go beyond simple collections of behavioral traditions, acquired through social learning, usually confined within small social units (e.g., Hirata et al., 2001; Muller and Cant, 2010), and rarely spreading into group-specific patterns. In contrast, culture with simple metarepresentational processes (re-representation) may be present in great apes, and this may have served as the evolutionary origins of another type of culture: a pattern of ideas that have normative force. Finally, culture with complex metarepresentation characterizes human culture, which is based on belief psychology, shared knowledge, values, and norms.

In this article, we focussed largely on chimpanzee tool use to illustrate how to analyze the cognitive aspects underlying animal culture, particularly the role of mental representations. We believe that our framework has a generic value and can be applied to all species with behavioral traditions, granted that they have the necessary brain structures for higher cognition, such as a neocortex (mammals) or equivalent structures in other species (for instance, the dorsal ventricular ridge in birds, Dugas-Ford et al., 2012), following Gärdenfors (2006). Although much of animal culture is material, there is evidence that a number of social behaviors also qualify as cultural (e.g., Whiten et al., 1999; Perry et al., 2003a; van Schaik et al., 2003), suggesting they make equally interesting candidates to study the role of representations, re-representations and metarepresentations.

In conclusion, to properly compare animal and human cultures it is necessary to identify the metarepresentational processes that underlie behavior. We have identified two types of metarepresentational processes. The first one is self-oriented, allowing an individual to access its own knowledge (‘re-representation’). The second one is based on mental state attributions, allowing an individual to have access to more efficient transmission of knowledge (‘metarepresentation’). How the two co-evolved will need consideration, notably as primate cognition evolved within stable social groups (Byrne and Whiten, 1988). Great apes are the only available model to assess the evolutionary transition in behavior and cognitive abilities from early hominins to modern humans (McPherron, 2013). It seems safe to assume that early hominins possessed material cultures at least as complex as described for modern great apes (van Schaik et al., 2003), but at what stage they also became aware of their own culture as one possible variant is impossible to decide. Progress can be made by targeted research on great apes and other animals, concerning their relationships with artifacts. A key test is whether an animal would be surprised if another group member deviated from an established technique to solve a familiar task. Current progress in infant and child cognition research offers promising new avenues, notably in adapting studies of object categorisation, representation, and conceptualisation (Kelemen and Carey, 2007; Mandler, 2007; DiYanni and Kelemen, 2008; Ruiz and Santos, 2013). For instance, research on functional fixedness will provide a deeper understanding of the cognitive underlay of primate culture. Here, the key prediction is that chimpanzees raised in captivity (with no experience in nest building) should solve the honey-trap experiment more easily than wild chimpanzees with no stick use tradition (such as the Sonso community).

It is possible that great apes have more advanced metarepresentational capacities than generally thought (Call and Tomasello, 2008), although they seem to be better described as re-representations (*sensu* Karmiloff-Smith, 1992). Building on previous work, we argued that apes may be able to search through their own cultural knowledge to select the most adequate tool for a given task, as described in **Figures 2B** and **3A**, but more research is needed to test this hypothesis. In particular, experiments are required in which apes need to maintain several representations simultaneously to succeed. While they are probably able to associate particular individuals with particular techniques (**Figure 3B**), it seems less likely that they can associate a given behavior with a group of individuals, a necessary condition to understanding culture as a shared property of minds. Again, specific experiments will be required to address this hypothesis. Finally, we find it implausible that apes are able to attribute cultural beliefs to members of their groups (**Figure 3C**), which would require complex metarepresentational abilities. Once the necessary studies have been conducted, it should be possible to draw more certain conclusions about the nature of the mental representations underlying animal culture, which is ultimately necessary to understand the evolution of the human cultural mind.

## Conflict of Interest Statement

The authors declare that the research was conducted in the absence of any commercial or financial relationships that could be construed as a potential conflict of interest.

## Footnotes

1 We argue that the ability to form metarepresentations enables an individual to engage in self-analyzing processes (see **Table 1**), an ability also termed ‘metacognition’ (Beran et al., 2012). In animals, metacognition is typically studied with uncertainty tasks, in which a subject may opt for a lower-value reward when it is unsure to obtain a large reward depending on how correctly it can assess its own state of knowledge (Beran et al., 2012). There is a debate on whether there is a true difference between metacognition and metarepresentation or whether the former is part of the latter. For instance, Carruthers (2009) regards metacognition as mindreading applied to the self (**Table 1**). The main point of debate is whether or not the content of metacognition is representational (Proust, 2007; Carruthers, 2009). In our case, we have focussed on the representational nature of apes’ knowledge, suggesting that metarepresentation is the more appropriate term.
